# Rhamnolipids as Effective Green Agents in the Destabilisation of Dolomite Suspension

**DOI:** 10.3390/ijms221910591

**Published:** 2021-09-30

**Authors:** Krzysztof Jan Legawiec, Mateusz Kruszelnicki, Anna Bastrzyk, Izabela Polowczyk

**Affiliations:** Department of Process Engineering and Technology of Polymer and Carbon Materials, Wrocław University of Science and Technology, Wybrzeże Wyspiańskiego St. 27, 50-370 Wrocław, Poland; mateusz.kruszelnicki@pwr.edu.pl (M.K.); anna.bastrzyk@pwr.edu.pl (A.B.); izabela.polowczyk@pwr.edu.pl (I.P.)

**Keywords:** hydrophobic coagulation, glycolipid biosurfactant, surface hydrophobicity, sedimentation

## Abstract

In this paper, we describe an application of mono- and dirhamnolipid homologue mixtures of a biosurfactant as a green agent for destabilisation of a dolomite suspension. Properties of the biosurfactant solution were characterised using surface tension and aggregate measurements to prove aggregation of rhamnolipids at concentrations much lower than the critical micelle concentration. Based on this information, the adsorption process of biosurfactant molecules on the surface of the carbonate mineral dolomite was investigated, and the adsorption mechanism was proposed. The stability of the dolomite suspension after rhamnolipid adsorption was investigated by turbidimetry. The critical concentration of rhamnolipid at which destabilisation of the suspension occurred most effectively was found to be 50 mg·dm^−3^. By analysing backscattering profiles, solid-phase migration velocities were calculated. With different amounts of biomolecules, this parameter can be modified from 6.66 to 20.29 mm·h^−1^. Our study indicates that the dolomite suspension is destabilised by hydrophobic coagulation, which was proved by examining the wetting angle of the mineral surface using the captive bubble technique. The relatively low amount of biosurfactant used to destabilise the system indicates the potential application of this technology for water treatment or modification of the hydrophobicity of mineral surfaces in mineral engineering.

## 1. Introduction

The mineral processing sector plays a critical role in local and global development. With scientific and technical progress, the demand for raw materials necessary for manufacturing different types of goods is increasing. In this context, it is necessary to modify the raw material sourcing strategy in a more environmentally sustainable way [1], for example by reducing the consumption of water and energy, as well as minimising the use of harmful chemicals necessary for industrial processes.

One of the problems is the removal of fine solid particles that are generated during the excavation and processing of minerals [2,3]. The removal of such particles dispersed in aqueous medium is often a challenging issue due to their relatively small diameter (and thus mass). Furthermore, these entities do not sediment spontaneously and their settling time is very long. Therefore, progress toward finding new solutions to this problem is on the borderline of several disciplines, including colloid science, water purification, and metallurgy [4].

For faster settling of fine solid particles, it is necessary to aggregate such particles to give them a higher mass in volume, which will contribute to higher migration velocities in dispersion. Coagulation, hydrophobic coagulation, or flocculation are used to perform the aggregation process. One of the processes, hydrophobic coagulation, represents a specific phenomenon since it is based on the modification of the hydrophobicity of the solid surface, allowing the forces of hydrophobic interactions to overcome dispersion-stabilising electrostatic repulsive force [5]. In order to obtain an adequate hydrophobicity of the surface, one possibility is to use surface active molecules that will change the properties of the surface through adsorption. In the literature, one can find research on the use of synthetic surfactants for this purpose (e.g., [6,7,8,9,10]). However, the environmental impact of these substances is often negative, and new alternatives to replace synthetic surfactants with more sustainable molecules are being explored. As a cheap and eco-friendly alternative, quaternary ammonium salts have been used to destabilise quartz [11], kaolinite and quartz [12] suspensions, and coal slurry [13]. Nonetheless, biosurfactants secreted extracellularly by microorganisms have lower toxicity and higher biodegradability and are additionally characterised by high stability under a wide range of conditions, such as pH, temperature, and presence of electrolytes [14]. Consequently, they can be applied to change the properties of solid surfaces in systems with different pH [15] or for environmental remediation [16] without losing their unique ability to change the interfacial properties [17,18]. Up to date, most research has focused on studies of surfactin analogues adsorption on the magnesite, serpentinite, silica particles [19], and coal [20]. However, only Augustyn and co-workers describe the mechanism of surfactin molecules adsorption onto coal particles as physisorption through hydrophobic interactions [20]. In the case of glycolipid biosurfactants, rhamnolipids (RLs), the surface of magnesite solid waste [21], and hematite particles [22] have been modified. However, these studies have not focused on achieving effective destabilisation of the dispersion systems and on the precise explanation of the mechanism of this process.

Here, we present for the first time the results of measurements of dolomite surface wettability in a rhamnolipid solution environment using the captive bubble method and the possibility of controlling the mineral suspension destabilisation by biomolecules. To gain insight into the ability of RLs to destabilise fine particle mineral suspension, we tested a mixture of mono- and di-RL homologues with regard to their ability to modify the surface hydrophobicity of a naturally hydrophilic carbonate mineral (dolomite). The properties of the biosurfactant solution were characterised by surface tension and aggregate size measurements. The adsorption isotherm of the biosurfactant on the dolomite surface was then determined. Changes in dolomite surface properties after rhamnolipid adsorption were confirmed by measuring the electrokinetic (zeta) potential. Sedimentation kinetics were determined by turbidimetry, and we reported for the first time migration velocity rates of dolomite particles in the presence of RL molecules using backscattering profile analysis.

## 2. Results and Discussion

### 2.1. Biosurfactant Molecules Behaviour in a 1 mM NaCl Aqueous Solution

**Figure 1:**
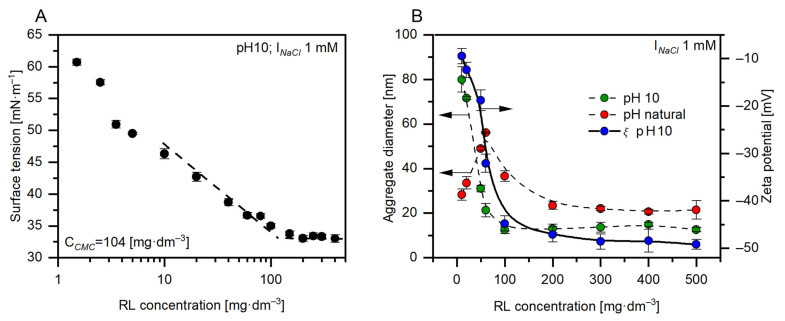
(**A**) Surface tension isotherm of RL aqueous solution (pH 10, 1 mM NaCl, 25 °C). The standard deviation for all the investigated samples was less than 0.6 mN·m^−1^. (**B**) Variation of hydrodynamic diameter (1 mM NaCl, 25 °C) combined with changes in zeta potential versus RL solution concentration (pH 10, 1 mM NaCl, 25 °C—blue data series).

Rhamnolipids (RLs) are surface-active compounds of biological origin structurally composed of one or two rhamnosyl groups joined with a *β*-fatty acids chain. The hydrophobic part of this biomolecule also contains carboxylic acid groups that dissociate depending on pH and ionic strength [23]. Bacteria produce RLs in a different proportion of different homologues [24]. In this study, a mixture composed mainly of mono-RL (rhamnose C10-C10) and di-RL (rhamnose-rhamnose C10-C10) was used. Current knowledge of the various RL homologues indicates that biomolecule homologues behave differently in both the solution and the interface [25], which complicates the theoretical prediction of specific surface-active properties [26,27]. Data from the literature show that examined glycolipids can reduce water surface tension to 40 or even 25 mN·m^−1^ (depending on the composition of the homologue mixture, purity, pH of the solution, and ionic strength) [28,29]. Therefore, to characterise a particular mixture of RL homologues, it is most convenient to determine the effectiveness of surface tension reduction. Surface tension measurements for the aqueous solution of the RLs mixture used in this paper are shown in Figure 1A. It can be seen that the surface tension was reduced from 72.0 mN·m^−1^ to 33.6 mN·m^−1^. The obtained results provided data for the determination of the critical micelle concentration (CMC) (Figure 1A). The CMC value for RLs aqueous solution at pH 10 with 1 mM NaCl was found to be 104 mg dm^−3^. According to literature reports, this parameter is in the range of 20–120 mN·m^−1^ depending on the pH and ionic strength [25,30,31,32]. The pKa value is 5.5. and 5.6 for mono- and di-RLs, respectively [33]. At pH above this value, the carboxylic groups in RLs molecule are in the majority deprotonated. Sodium cations originating from the electrolyte can shield the negative charge [34]. In effect, more packaged structures of biomolecule aggregates can be formed, which decrease CMC values compared to systems without added ions [24,32,35,36]. However, in our case, the CMC value (104 mg·dm^−3^ at 1 mM NaCl; Figure 1A) is high despite the presence of the electrolyte because the rhamnolipid molecules are fully deprotonated at a strongly alkaline pH [23]. Thus, more negatively charged molecules exhibit a greater intermolecular repulsive force, and the molecules form micelles at higher concentrations [35]. We suppose that the Na^+^ concentration is too low in the tested system to reduce the repulsion between the molecules.

The very idea of using CMC as a parameter to determine the formation of micellar aggregates is more applicable to single-chain surfactant solutions. As is known, RL is a double chain surfactant; therefore, aggregation occurs already at a lower concentration, called critical aggregation concentration (CAC) [28,36]. In the literature, reports can be found that aggregation occurs at one-tenth of the CMC concentration [37]. To evaluate spontaneous aggregation of RLs molecules, the size of the aggregates was determined using the DLS technique. It is known that RLs can be organised in various forms, such as lamellar, vesicles, and micelle [38,39]. Comparative analysis of system self-assemblement was performed at natural pH (7.5 ± 0.4) and pH 10. The evolution of aggregates size with increasing concentration was shown in Figure 1B (red and green data series). As can be seen, depending on the pH, different natures of changes in aggregate size can be observed. This results from different deprotonation of RL molecules in aqueous media of different pH [40]. For a solution at natural pH, we initially observed a gradual increase in aggregate size from 28 nm in a solution with a concentration of 10 mg·dm^−3^ RL, up to 56 nm in a solution with a concentration of 60 mg·dm^−3^. Subsequently, the size of the aggregates decreases to about 20 nm at higher concentrations. The CAC is distinctively visible. This phenomenon is related to the lack of control of the pH of the system [32]. At pH 10, it can be observed that as the concentration of RL solution increases, the average hydrodynamic diameter (aggregate diameter) decreases from 80 nm to 12 nm after reaching CMC (104 mg·dm^−3^), which correlates with an increase in the absolute value of the zeta potential; therefore, the micellar aggregate is more negatively charged. RL solution zeta potential measurements were also performed at pH 10 (Figure 1B, blue data series). The measured average result of −44.8 ± 1.6 mV for concentrations greater than two times more CMC is consistent with previous studies by Haryanto and Chang [41]. For the concentration value near CMC, the zeta potential was −18.8 ± 2.2 mV. The visible breakthrough of the trend is observed when the concentration of biomolecules approaches CMC. This can suggest that molecule aggregates became more negatively charged with increasing RL concentration, which is typical for anionic-like surfactants [42].

### 2.2. Adsorption Isotherm of Rhamnolipids on the Dolomite Surface

RL as surface active agents have the ability to self-organise not only at the water–air interface but also at the solid–water interface. Therefore, the tested concentration of RL was in the range of 5 to 6000 mg·dm^−3^ to investigate all possible surface modification mechanisms that would be useful to predict suspension stability. It can be assumed that RL biomolecules can behave similarly to synthetic anionic surfactant molecules on the solid surface, based on earlier reports [43].

**Figure 2:**
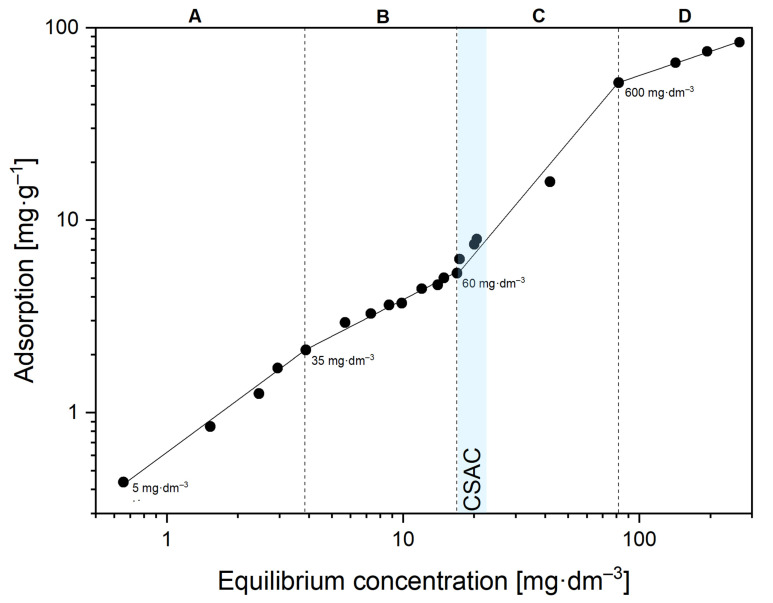
Rhamnolipid adsorption isotherm onto dolomite surface at pH 10. The initial concentration of the RL solution (5, 35, 60, 600 mg·dm^−3^) at the beginning of each surfactant adsorption region (**A**–**D**) was depicted. CSAC (critical surface aggregation concentration) in this system occurs at around 60–75 mg·dm^−3^ of the initial RL concentration.

The adsorption isotherm of rhamnolipid biomolecules on dolomite particles is presented in Figure 2. Due to its characteristic shape, it can be said that the isotherm corresponds to the classical surfactant adsorption model developed by Somasundaran and co-workers [44], in which four distinct regions occur. At concentrations of 5–35 mg·dm^−3^ (first stage of adsorption, Figure 2A), adsorption is based on electrostatic interactions between a single biomolecule RL and an oppositely charged surface. Then, in the second stage of adsorption (Figure 2B), there is a slight change in the isotherm slope. In this range of initial RL concentration (55–60 mg·dm^−3^), additional interactions occur between aliphatic chains in the hydrophobic portion of the biosurfactant (lateral interactions) [45]. In this case, the surfactant molecules adsorb with the tails facing the solution, making the mineral surface hydrophobic. In the description of region II adsorption, according to Somasundaran and Fuerstenau’s theory, there is a sharp increase in adsorption, which is explained as the effect of the formation of two-dimensional aggregates called solloids—surface colloids (e.g., hemimicelles). Despite limited papers on surface active biomolecules adsorption on solids, a similar second region of adsorption efficiency has been shown for surfactin analogues adsorbed on the silica, magnesite and serpentinite surface [19], and for rhamnolipid molecules adsorbed in soil [46], but has not been extensively analysed. A further increase in concentration leads to the formation of other aggregates, admicelles (Figure 2C). The formation of admicelles on the solid surface is related to the orientation of the surfactant. The hydrophilic heads are oriented both to the surface and facing the solution [44]. At this stage, the dolomite surface is electrically neutralized by RL molecules. The concentration at which admicelle structures are formed is termed critical surface aggregation concentration (CSAC) and typically occurs at a concentration of about 60% CMC [47]. When CMC is reached in the bulk of the RL solution, the flattening of the fourth adsorption region can be observed (Figure 2D). The surface is covered with a multilayer of biomolecules.

**Figure 3:**
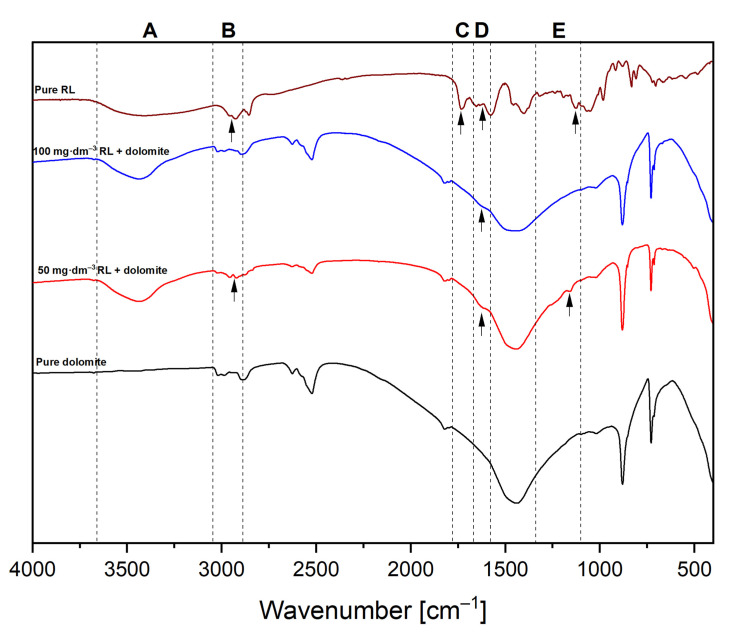
FT-IR spectra of pure RL, dolomite, and sediment obtained after contact with aqueous solutions of RL of concentrations of 50 and 100 mg·dm^−3^. (**A**–**E**) Identified regions used to describe the RL adsorption process on the dolomite surface.

FT-IR spectra were recorded to complement the rhamnolipid adsorption studies. As shown in Figure 3, we noticed five characteristic regions where different bands appear, which is more evidence of dolomite surface modification. In the range of 3600 to 3100 cm^−1^ (Figure 3A), the specific signals can be observed for a sample of RL and dolomite with adsorbed biosurfactant. This is an effect of -OH stretching of hydroxyl groups present in the surfactant molecule. There is no intensity fluctuation for the pure dolomite sample. The mineral structure does not have hydroxyl groups [48]. Another band is located at ~3000 cm^−1^ (Figure 3B) and is related to the aliphatic stretching of -CH and methylene stretching (-CH_2_) [49]. This band is only visible for the sample treated with 50 mg·dm^−3^ of RL. For a sample with an initial RL concentration of 100 mg·dm^−3^ there is no band of sufficient intensity, which can be considered as an effect of the formation of a double layer on the mineral surface (Figure 2D, the surfactant molecule faces the polar heads in the bulk of the solution). The presence of an ester bond (stretching vibrations of -C=O, Figure 3C) from the ester functional group at ~1600 cm^−1^ is demonstrated only for the pure rhamnolipid sample. The disappearance of the peak in this band for samples after adsorption indicates that complexation of the metal bi-positive cations with biomolecules occurred, and thus the bond decayed. The D band (Figure 3D) shows signals from the carboxyl group with a different intensity (bending of -O-H in bands in the -COOH group) [50]. The peaks in the E range (Figure 3E) are the effect of the C-O-C stretching in the rhamnose [51] present in the hydrophilic part of the molecule.

To understand and describe in detail the adsorption mechanism of a rhamnolipid molecule, which is structurally different from the synthetic anionic surfactants of relatively simple morphology discussed in the literature to date, it is necessary to analyse the molecular phenomena that may take place at the phase boundary and its vicinity. In the rhamnolipid molecule, regardless of the homological structure (presence of rhamnose groups in mono- and di-RL), specific rotation occurs between the hydrophilic and hydrophobic parts of the biosurfactant because the bond that connects two regions of the molecule is flexible [52]. Sugar-originated hydrophilic heads have a different positive charge density in the regions around hydrogen atoms; then, adsorption of the hydrophilic portion of the surfactant molecule is strictly related to rhamnosyl group adsorption on the mineral surface via hydrogen bonding. Another mechanism of adsorption of the RL molecule is surface complexation and ion exchange reaction [46]. All interactions leading to complexation are possible through the carboxyl and carbonyl groups in the hydrophobic part of the molecule and the oxygen atom between the hydrophobic and hydrophilic parts of the biomolecule [52]. In the case of the dolomite–water interface, the findings related to the binding of metal cations by biosurfactant molecules can be sufficient to explain the interactions between calcium and magnesium ions, which are the components of the dolomite mineral [46]. Several works can be found in the literature in which metal ions were chelated by rhamnolipids or other biosurfactants [53,54,55,56]. This leads to the formation of [Me^2+^]-RL complexes. From energy-minimised molecular mechanics structures, it was proven that the distortion was more significant after incorporating a divalent metal cation than for the sole rhamnolipid molecule [57].

**Figure 4:**
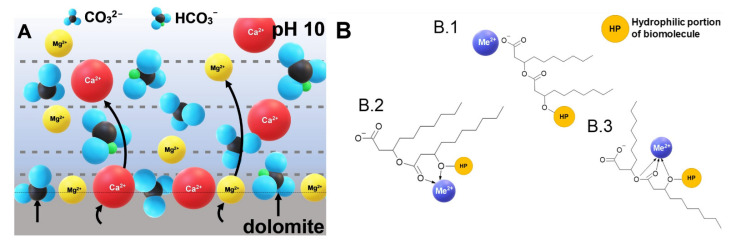
(**A**) The state of the surface and ionic forms of dolomite released to water at pH 10; (**B.1**–**B.3**) examples of [Me^2+^]-rhamnolipid complexes based on [52] at pH 10.

The carboxyl group binds calcium or magnesium cations released from the dolomite rock, forming an ionic complex (Figure 4B.1). Formation of bi- (Figure 4B.2) and tri- (Figure 4B.3) coordinated complexes of metal cations with rhamnolipid anion is also possible [52]. Dolomite is a sparingly soluble salt-type mineral, so various forms of lattice ions originating from cation hydrolysis and anion proton reaction can be identified in the aqueous environment [58]. Dolomites are naturally hydrophilic minerals, unstable at acidic pH due to their chemical nature, and can buffer the solution to a specific pH value around 10 (in conditions of pH < 7, mineral particles slowly decompose by dissociation) [59,60]. Ca^2+^ and Mg^2+^ cations are released from the mineral surface, but at pH 10 their concentration is much lower than the concentration of anions, such as HCO_3_^−^ and CO_3_^2−^ [61] (Figure 4A).

### 2.3. Stability of Dolomite Suspension in the Presence of Rhamnolipids

**Figure 5:**
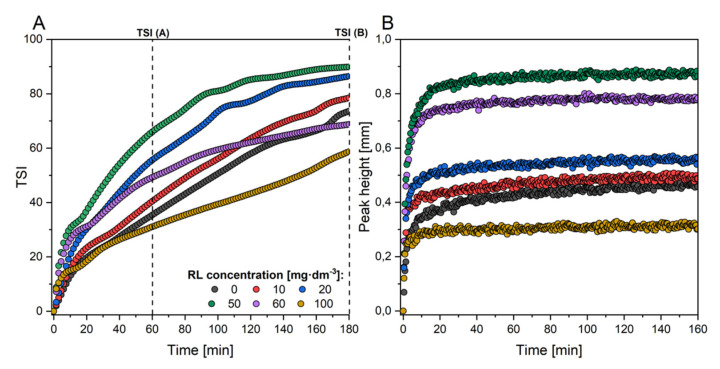
Kinetics of destabilisation of the dolomite suspension (**A**). Peak height curves that define the relationship between sediment thickness and the time over which it increases (**B**).

The destabilisation kinetics of the dolomite suspension at various concentrations of RL are depicted in Figure 5. Figure 5A shows the changes of the Turbiscan Stability Index (TSI) as a function of time. The combination of TSI points over time gives the complete destabilisation kinetics of the investigated suspension [62]. The TSI values after 60 (A) and 180 (B) minutes for the investigated system are presented in Table 1.

From the general evaluation of the results of the turbidimetric analyses, it can be seen that at low concentrations of biosurfactant (below CMC), aggregation of dolomite occurs at a more significant level, resulting in an increased rate of sedimentation. The reason may be that the RL molecule behaves similarly to gemini surfactants. Therefore, aggregate formation occurs much below the CMC [37,63], which we have proven through DLS measurements (Figure 1B). At higher RL concentrations (near CMC), an elevated stabilisation can be observed. The instability index for the dolomite suspension without biosurfactant was 35.4 and 73.6 after 60 and 180 min, respectively. Such values suggest that dolomite particles are suspended in the solution. As shown in Table 1, the zeta potential value measured for the system without added RL (pH 10 and 1 mM NaCl) was −6.3 mV, which suggests that the dolomite suspension is unstable, but the migration velocity rate is relatively slow (6.66 mm·h^−1^).

**Figure 6:**
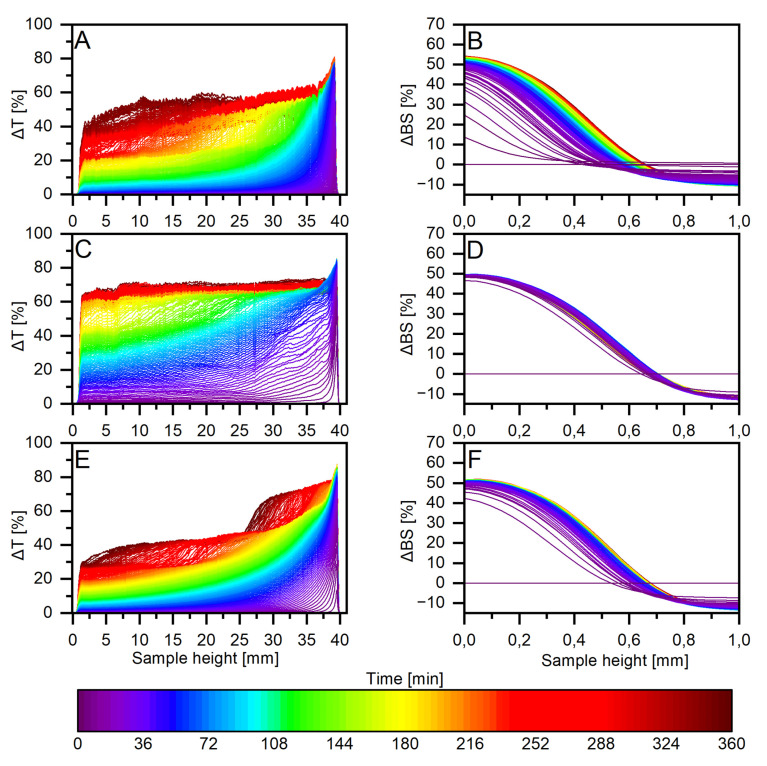
Transmittance profiles ((**A**), control sample, (**C**), 50 mg·dm^−3^ RL, (**E**), 100 mg·dm^−3^ RL) and backscattering ((**B**), control sample, (**D**), 50 mg·dm^−3^ RL, (**F**), 100 mg·dm^−3^ RL) for dolomite suspensions.

However, if one looks at the curves representing the sediment growth rate over time (Figure 5B), it can be seen that the TSI parameter does not always correlate with the migration velocities obtained (Table 1). This is related to the fact that the value of the TSI parameter at a particular point in time is the cumulative sum of all the backscattering or transmission variation of the entire sample. Regarding the migration velocity values, these are calculated only from the evolution of backscattering profiles (Figure 6B,D,F; the methodology for calculating migration velocities is described in Section 3.4).

It can be seen that the migration velocity increases with increasing biosurfactant concentration but up to a limit value, after which the system becomes stable, and the migration velocity values decrease. The critical point for increasing this parameter is the concentration of 50 mg·dm^−3^, for which the migration velocity was calculated as 20.29 mm·h^−1^. For systems where the concentration of RL was higher, a continuous decrease in the value of the velocity parameter was observed (up to 10.99 mm·h^−1^ for a suspension with 100 mg·dm^−3^ of biomolecules).

**Figure 7:**
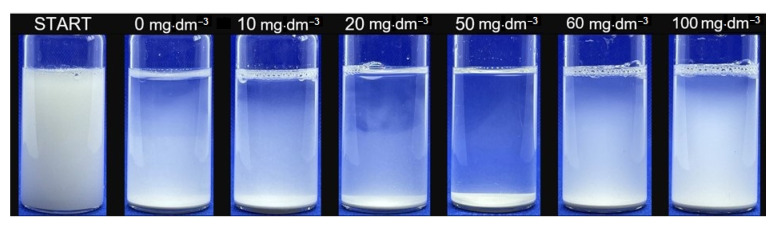
Dolomite suspension with different doses of RL at the beginning (START) and after 180 min of sedimentation. In the sample with 50 mg·dm^−3^ of RL, the most transparent solution can be observed.

The addition of biomolecules to the investigated system significantly affects the suspension behaviour. Under low concentrations of RL, the system becomes more unstable, as shown in Figure 7. It can be deduced from Figure 5A that in the concentration range from 10 to 20 mg·dm^−3^, the TSI value measured in time has similar values (TSI at 180 min: 84.1–86.4), which is related to the formation of the monolayer of a biomolecule on the solid surface. There is a sharp increase in destabilisation (TSI 89.9 at 180 min), which means that coagulation and sedimentation processes have occurred. This value is extreme in the examined system, which means that the fastest destabilisation can be observed with a relatively low concentration of RL, amounting to 50 mg·dm^−3^. By analysing the transmittance profiles, it can be seen that, despite the natural instability of the dolomite suspension, the clarification of the solution (reaching high transmittance values) occurs much faster after the addition of biosurfactant (Figure 6A,C), while for a system in which a relatively high concentration of RL is present, an evident stabilisation occurs (Figure 6E).

This work investigated the stability of an aqueous suspension of fine dolomite particles at pH 10. Due to the different organisation of RL molecules on the mineral surface, different aggregation mechanisms and stabilisation of the colloidal system can be expected [7]. For two particles to form an aggregate, the thin liquid film formed between them must be ruptured. According to the DLVO theory, the stability of this film (and thus the colloidal system) is affected by the repulsive forces of the electric double layer and the attractive van der Waals forces [64]. In our mineral suspension, the magnitude of these interactions was modified by the adsorption of surface-active substances of anionic nature. However, these forces are not the only ones that interact in a system with surfactants. Several theoretical and experimental studies [65,66,67] provide information that in such systems there are additional forces, referred to as hydrophobic forces, which affect the stability of the film and can be the source of so-called hydrophobic coagulation. The contribution of these forces increases as the hydrophobicity of the surface increases, causing these forces to predominate over the repulsive electrostatic forces once a certain degree of hydrophobicity (expressed by the contact angle) is exceeded.

One of the most comprehensive pieces of evidence for the dolomite aggregation mechanism is the analysis of RL’s ability to hydrophobise the surface. Several methods are available to determine the influence of adsorbed surfactant on the wettability of the mineral surface, including measuring the contact angle by the sessile drop method [68,69]. In our system, the best correlation between surface wettability and turbidimetric measurements of suspension stability was obtained using the captive bubble method to measure the contact angle. This method represents conditions closer to those in aqueous solution, where the mineral surface is immersed in liquid, than the sessile drop technique where a water drop is placed on a dry sample. In general, surface hydrophobicity studies provide much important information on the dispersion system and are crucial to predicting its stability [69].

Table 2 shows the results of the surface wettability measurements performed for polished mineral rocks immersed in RL solutions of concentration in the range of 0–1000 mg·dm^−3^. It can be seen that the rate of sedimentation of the mineral particles varies according to the measured contact angle. With increasing RL concentration (in the range of 0 to 50 mg·dm^−3^), the surface coverage of dolomite increases. The adsorbed biosurfactant forms a monolayer on the surface of the mineral particle, with the head adhering to the surface and the tail facing the solution, making the surface more hydrophobic. With increasing hydrophobicity, the hydrophobic acting forces are more significant and predominate over the repulsive electrostatic forces. Therefore, the sedimentation rate increases with increasing RL concentration, despite a simultaneous increase in the absolute zeta potential value (Table 1), resulting from the adsorption of the anionic surfactant. The increase in potential values should influence the suspension’s stabilisation; however, a destabilisation of the suspension is noticeable. The maximum of these interactions can be seen at 50 mg·dm^−3^, where the zeta potential was equal to 21.5 mV, and the wetting angle has the highest value of 36°, which means that the highest surface hydrophobisation was obtained. Coagulation occurs at relatively high absolute values of the zeta potential of 21.5 mV (Table 1), suggesting that hydrophobic interactions drive it. The zeta potential for the reference sample (without RL) was −6.3 mV, which means that sedimentation should occur faster than for a concentration of 50 mg·dm^−3^, but no such effect was observed. When the concentration of 50 mg·dm^−3^ is exceeded, based on data from adsorption isotherms, it can be seen that a second surfactant layer forms on the surface of the particle, in which this time it is the heads that are directed toward the solution, which translates into an increase in the negative zeta potential to ca. −30 mV, and decrease in surface hydrophobicity and thus stabilisation of the suspension. It is known based on the literature that after exceeding the absolute value of the zeta potential of 30–40 mV, the colloidal aqueous solution may become stable [70,71].

It can be concluded that there is an experimental correlation between these properties, which is measurable by the methodology presented. Hydrophobic coagulation occurs as long as the surface hydrophobicity increases with the increasing concentration of the hydrophobisation agent [6]. Therefore, it can also be concluded that the contact angle to determine the dominant hydrophobic interactions (hydrophobic coagulation) over electrostatic repulsion is the value of 36° reached for a concentration of 50, which reflects the conformation of the surfactant molecules on the mineral surface (hemimicelles formation) which is consistent with the breakthrough between the II and III regions of the adsorption isotherm (Figure 2B,C). For higher concentrations in the range of CSAC, a change in zeta potential can also be observed (from −21.5 mV for 50 mg·dm^−3^ to approximately −30 mV for 60 mg·dm^−3^, Table 1). As a result of interchain interactions and the formation of bilayer structures, the surface becomes more hydrophilic (Figure 2C,D), which was also proven by contact angle measurements (the value of contact angle 21 ± 2 deg., Table 2).

As mentioned above, the stability of the suspension changes as a result of the hydrophobic interaction induced by the addition of biosurfactant to the suspension. A faster settling of particles is possible after their aggregation when the density of the aggregate is greater than the density of the medium in which the particle is dispersed. The explanation for this phenomenon lies in the sizes of the aggregates, which were studied by the light scattering technique. Table 3 shows the parameters of the size distributions and the calculated fractal dimension of the aggregates of dolomite particles in the presence of RL.

The dose of RL in dolomite suspensions used for laser diffraction measurements was the same as in stability experiments. Fairly significant changes in size distribution are observed when more than 50 mg·dm^−3^ biosurfactant is added to the system. Evidently, at this concentration, the most efficient aggregation is obtained and, as confirmed in the sedimentation experiment, has the fastest particle settling. It should be noted here that the polydispersity index does not change significantly. The relatively high mean PDI value (3.5) according to the Mercus classification [72] indicates the average heterogeneity of the suspension, as can be seen in Figure 7, where the finest fractions remain suspended in solution, while the largest fractions fall to the bottom of the measuring cell. The higher the value of the fractal dimension, the more complex and condensed the aggregate structure. The fractal dimension of the aggregates obtained by hydrophobic coagulation also has the highest value for 50 mg·dm^−3^ of added biosurfactant.

**Figure 8:**
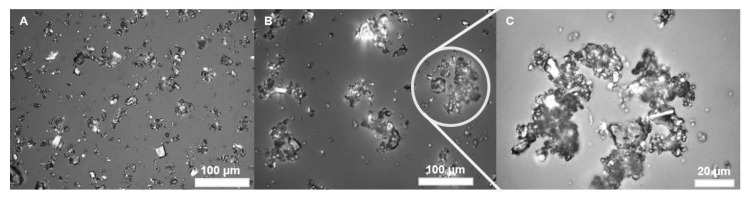
Optical microscope images of the dolomite sample (**A**), dolomite after coagulation with 50 mg·dm^−3^ RL solution (**B**), and a zoom in on the aggregate (**C**). The magnification of samples A and B was 40× and 100× for sample C, respectively.

Without the addition of RL, the dolomite particles are slightly aggregated (Figure 8A), but after the addition of a sufficient amount of biosurfactant (50 mg·dm^−3^), noticeable coagulation occurs due to hydrophobisation of the surface of the particle (Figure 8B,C). The structure of the aggregates becomes more complex, as confirmed by calculations of the fractal dimension and its increase from 1.28 to 2.10, which is also noticeable in the change of deciles of the size distribution (Table 3).

## 3. Materials and Methods

### 3.1. Biosurfactant Aqueous Solution Characterisation

Rhamnolipids (RLs) were obtained from AGAE Technologies LCC (>90% content of mono- and di-RL, Corvalis, OR, USA). The digital tensiometer T-10T (Krüss, Hamburg, Germany) equipped with a water thermostat coat that maintains temperature stability of ±0.2 °C was used to measure the surface tension of the RLs aqueous solution. Each measurement was performed three times. A validation procedure using ultrapure water was performed before every solution measurement. The value of critical micelle concentration (CMC) and surface tension at CMC was calculated from the intersection of two straights drawn in low and high concentration regions of surface tension curves (Figure 1A) using the least squares method. Micellar aggregate size measurements were made using the Photocor Complex light scattering spectrometer system (Photocor, Tallinn, Estonia) with a diode laser (657 nm, scattering angle 90⁰) for RL concentrations, such as 10, 20, 50, 60, 100, 200, 300, 400 and 500 mg·dm^−3^ providing a constant ionic strength (1 mM NaCl) and pH around 10. All reagents were used without further purification. The water was supplied by the Millipore Simplicity-UV purification system (Merck, Darmstadt, Germany) with a conductivity of 0.05 μS·cm^−1^.

### 3.2. Mineral Sample Characterisation

**Figure 9:**
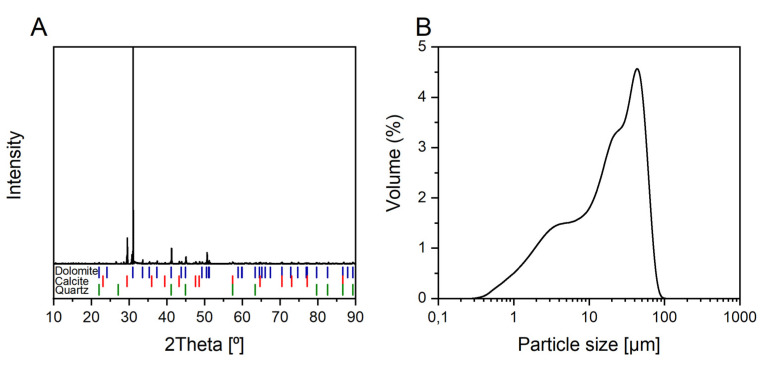
XRD pattern (**A**) and particle size distribution (**B**) of Kletno dolomite sample.

Dolomite CaMg(CO_3_)_2_ was obtained from an old quarry in Kletno (Poland). The preparatory procedure of the mineral sample included crushing dolomite rocks in a ceramic ball mill and fractionating in sieves. To perform an adsorption and stability study, a fine dolomite fraction of −40 µm was used. The particle size distribution was tested using a Mastersizer 2000 laser diffractometer equipped with a HydroMU2000 dispersion unit (Malvern Instruments Ltd., GB) and is shown in Figure 9B. Table 4 provides information on the dolomite fraction used, especially on the size distribution, such as the median diameter, d_50_, and the first and ninth deciles (d_10_ and d_90_). These diameters correspond to a diameter that does not exceed 50, 10 and 90% of the population, respectively. For the investigated dolomite mineral samples, d_10_, d_50_, d_90_ was found to be approximately 2.3, 19.3 and 51.3 µm, respectively. The specific surface area of dolomite particles was measured using the FlowSorbII (Micromeritics Instruments Corp. Norcross, USA) apparatus by the flowing gas BET method through adsorption and desorption of the helium–nitrogen mixture. A specific surface area (*S*_BET_) of the material was found to be 1.92 m^2^·g^−1^. The density of dolomite particles was determined using a pycnometer at 20 °C. The measurements were repeated three times, and the density was found to be 2.8 ± 0.2 g·cm^−3^. XRD analysis using Ultima IV powder diffractometer (Rigaku, Japan) was performed to confirm the purity of the sample. The sample is revealed to consist of high purity dolomite with an insignificant amount of quartz and calcite (Figure 9A).

### 3.3. Adsorption Isotherm of Rhamnolipid onto Dolomite Particles

A four-step procedure was used to prepare the dolomite suspension in a rhamnolipid aqueous solution. First, 0.2 g of dolomite powder was placed in a glass vial. After that, an adequate volume of Mili-Q water was added and sonicated to disperse the particles. Next, the calculated amount of RL solution and sodium chloride as background electrolyte (1 mM) was added, obtaining samples with biosurfactant concentrations from 0 to 6000 mg·dm^−3^. The total suspension volume was 20 cm^−3^, and the pH was adjusted to a value of approximately 10 using sodium hydroxide solutions (0.1 and 0.01 M) of analytic purity. The dolomite suspension was mixed overnight to equilibrate at a constant temperature using a magnetic stirrer (25 °C, 250 rpm). After this time, the suspensions were centrifuged, and the supernatant was used for the determination of the RL content using a modified procedure proposed by Dische and Schettles [73]. Briefly, 11 aliquots of RL were made with a concentration range from 4.0 to 40 µg·cm^−3^ RLs to prepare the calibration curve. Then 4.5 mL of a 9.45 M sulfuric acid solution was added to the test tubes containing 1 mL of each RL solution. After this sequence, the tubes were placed in boiling water for 10 min. The RL solutions were then cooled and thioglycolic acid (Sigma-Aldrich, >98% purity) was added. All tubes were kept in the dark for three hours. The absorbance of the prepared RL solution samples was measured at 400 nm against the blank (diluted sulfuric acid) using a UV-VIS spectrophotometer (Evolution 210, Thermo Scientific, Waltham, MA, USA). To determine the concentration of residual biomolecules in the solution after the sorption process, 1 mL of each supernatant was tested following the calibration curve procedure. All measurements were made twice and an average value was reported. The amount of biosurfactant adsorbed onto the surface of the dolomite particle was determined by using the depletion method.

To prove the chemical interactions between RL molecules and a mineral surface, FT-IR spectra for pure dolomite and pure RL were recorded and compared with sediment obtained after centrifugation of suspension consisting of 50 and 100 mg·dm^−3^ of RL.

### 3.4. The Dolomite Suspension Stability and Turbidimetric Analysis

Stability measurements of the dolomite suspensions were investigated using Turbiscan Lab Expert (Formulaction, Toulouse, France) based on the measured changes in turbidity. The device is equipped with two detectors, moving axially along a measuring cell made of glass, which measures the change of solid concentration and/or particulate size in dispersion by light backscattering (BS) as well as clarification of suspension by measuring light transmittance (T), both at 880 nm. The scanning is carried out with high resolution throughout the height of the measuring cell, every 40 µm. With this method, the TSI (Turbiscan Stability Index) could be estimated. Thanks to this parameter, one can assess whether suspension is stable (when TSI is close to 0) or not. TSI is a characteristic parameter calculated by an integration algorithm that sums up the evolution of light transmitted and backscattered over time [62]. The dolomite suspension samples were prepared according to the procedure described in Section 3.3. The total volume of suspension in the measurement cell was 20 cm^−3^. The duration of each analysis was 3 h and a single scan took 25 s. The interval between each subsequent scan also lasted 25 s. Solid phase migration rates were estimated by analysing backscattering profiles for the examined suspensions (Figure 6B,D,F). The group of curves that begin on the left-hand side of the backscattering diagrams provides information on how fast the sediment layer grows. Mathematical transformations produce a series of data that correlate the value of the sediment height in the measuring cell (peak height in mm) and the time at which this height changes (time in min) (Figure 5B). The migration velocity of the solid particles can then be determined from these data by linear approximation of the initial points of the peak height—a time data series. The equation describing the rate of change was defined by linear regression. From this equation, the slope value was taken, which is the parameter of interest. In the calculation process, satisfactory values of the solid phase particle migration velocity were considered those for which the coefficient of determination (R^2^) was at least 0.90. The result of this analysis as migration velocity in mm·h^−1^ was shown in Table 1.

### 3.5. Contact Angle Measurements

The contact angle of the water on the dolomite was determined by a captive bubble method using an optical goniometer OCA15EC (DataPhysics Instruments, Filderstadt, Germany). First, the dolomite rock samples were polished and immersed in RL solution (0, 10, 50, 60, 100 and 1000 mg·dm^−3^; 1 mM NaCl, pH 10). The samples were kept overnight until adsorption equilibrium was achieved. The dolomite mineral sample was then mounted in a PTFE holder and placed in a glass cell (40 mm × 40 mm × 80 mm) filled with an adequate concentration RL solution. The air bubble of 1 mm average diameter was generated using a 100 µL gastight syringe (Hamilton, OH, USA) equipped with an upward bent dosing needle. The images of the gas bubble were captured up to 10 s after reaching the bottom of the surface of the mineral sample and then analysed using SCA 20 software (DataPhysics Instruments, Germany) to determine the contact angle. This procedure was repeated ten times for each RL concentration, and the average value was calculated.

### 3.6. Zeta Potential Measurements

The zeta potential of RLs solution with 1 mM NaCl, pH 10 and dolomite particles dispersed in an aqueous solution of RLs (1 mM NaCl, pH 10) was measured using the Zetasizer 2000 apparatus (Malvern, UK). The suspension samples were prepared according to the procedure described in Section 3.4. Each measurement was carried out in a set of five repetitions.

## 4. Conclusions

In summary, mineral suspensions consisting of dolomite and RL were prepared and characterised. The conclusions of this study are as follows:

1. The most effective destabilisation of the dolomite suspension occurred when 50 mg·dm^−3^ RL was added to the dispersion of dolomite. Through the complexation of RL molecules with Ca^2+^ and Mg^2+^ ions on the mineral surface and hydrogen interactions with the hydrophilic part of the biomolecule, adsorption occurred. Covering the surface of the hydrophilic mineral with a surfactant provided effective surface hydrophobisation at this concentration.
2. The use of biomolecules makes it possible to control the size of the fine particle aggregates and their migration velocity rate in the aqueous solution. For the dolomite sample (fractal dimension was 1.28 and migration velocity was calculated at a level of 6.66 mm·h^−1^), these parameters were much lower than for the system with 50 mg·dm^−3^ of RL added (fractal dimension: 2.10 and migration velocity as 20.29 mm·h^−1^).
3. From the evolution of backscattering and transmittance profiles over time, the destabilisation index (TSI) was calculated and showed that it is possible to change the TSI from approximately 70 to 90 with an appropriate dose of biosurfactant. Exceeding the critical dose resulted in a decrease in the TSI value of the examined system to approximately 60 after 3 h.
4. Hydrophobic coagulation was proven using the captive bubble technique founded on the contact angle value of 36°.

The performed experimental results conclude that RL can significantly change the hydrophobicity of hydrophilic mineral surfaces, so it can be applied as surface modifiers in the mineral engineering industry and water treatment processes.

## Figures and Tables

**Table 1:** Changes in TSI, calculated solid phase migration velocity and zeta potential values for different RL concentrations. TSI (A) after 60 min and TSI (B) after 180 min. The results correspond to the curves in Figure 7 and Figure 8 accordingly.

RL Concentration [mg·dm^−3^]	0	10	20	50	60	100
TSI (A)	35.4	50.5	55.6	66.0	49.3	31.0
TSI (B)	73.6	84.1	86.4	89.9	68.7	58.7
Migration velocity [mm·h^−1^]	6.66	12.60	14.48	20.29	18.44	10.99
Zeta potential [mV]	−6.3 ± 0.6	−10.6 ± 0.9	−14.5 ± 0.9	−21.5 ± 0.4	−29.9 ± 0.2	−31.8 ± 0.9

**Table 2:** The contact angle (measured through the aqueous phase) of the dolomite surface measured in RL solutions of different concentrations.

RL Concentration	Captive Bubble Angle	
mg·dm^−3^	deg.	
0	16 ± 1	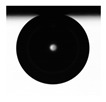
10	27 ± 2	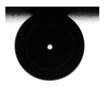
50	36 ± 1	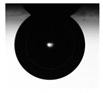
60	34 ± 1	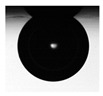
100	28 ± 2	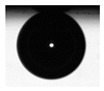
1000	21 ± 2	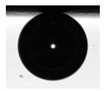

**Table 3:** Deciles of the particle size distribution (d_10,50,90_) with a polydispersity index (PDI) and fractal dimension (DF) values for dolomite samples contacted with various concentrations of RL.

RL Concentration	d_10_	d_50_	d_90_	PDI	DF
mg·dm^−3^	μm				
0	1.7	11.2	41.6	3.5	1.28
10	1.7	12.3	41.7	3.2	1.29
20	1.9	11.1	42.0	3.6	1.31
50	2.4	13.3	48.4	3.4	2.10
60	2.3	11.3	43.5	3.6	2.01
100	2.1	11.3	43.4	3.6	1.99

**Table 4:** Physical properties of dolomite sample. *S*_BET_—specific surface area; d_10,50,90_—deciles of size distribution.

*S*_BET_	d_10_	d_50_	d_90_	Density
m^2^·g^−1^	µm			g·cm^−3^
1.92	2.3	19.3	51.3	2.8

## Data Availability

Data available on request.
